# Marine Microalgae as a Nutritive Tool to Mitigate Ruminal Greenhouse Gas Production: In Vitro Fermentation Characteristics of Fresh and Ensiled Maize (*Zea mays* L.) Forage

**DOI:** 10.3390/vetsci10090556

**Published:** 2023-09-04

**Authors:** Mona Mohamed Mohamed Yasseen Elghandour, Aristide Maggiolino, Edwin Rafael Alvarado-Ramírez, Javier Hernández-Meléndez, Raymundo Rene Rivas-Cacerese, Pedro Enrique Hernández-Ruiz, Ameer Khusro, Pasquale De Palo, Abdelfattah Zeidan Mohamed Salem

**Affiliations:** 1 Facultad de Medicina Veterianria y Zootecnia, Universidad Autónoma del Estado de México, Toluca 50000, State of Mexico, Mexico; mmohamede@uaemex.mx; 2 Department of Veterinary Medicine, University of Bari A. Moro, 70010 Valenzano, Italy; pasquale.depalo@uniba.it; 3 Unidad Académica Multidisciplinaria Mante, Universidad Autónoma de Tamaulipas, El Mante 89840, Tamaulipas, Mexico; win.rmz@hotmail.com; 4 Facultad de Ingeniería y Ciencias, Universidad Autónoma de Tamaulipas, Ciudad Victoria 87149, Tamaulipas, Mexico; javhernan@docentes.uat.edu.mx; 5 Instituto de Ciencias Biomédicas, Universidad Autónoma de Ciudad Juárez, Ciudad Juárez 32310, Chihuahua, Mexico; rrivas@uacj.mx; 6 Escuela Superior de Medicina Veterinaria y Zootecnia No. 3, Universidad Autónoma de Guerrero, Técpan de Galeana 40900, Guerrero, Mexico; phernandezr007@alumno.uaemex.mx; 7 Research Department of Plant Biology and Biotechnology, Loyola College, Chennai 600034, Tamil Nadu, India; armankhan0301@gmail.com

**Keywords:** biogas, greenhouse gas, maize genotypes, *Dunaliella salina*, sheep

## Abstract

**Simple Summary:**

Human population growth is expected to increase the demand for livestock-derived food over the next three decades, and consequently, the emission of greenhouse gases, including methane (CH_4_), carbon monoxide, and hydrogen sulfide (H_2_S), will increase. Given this situation, microalgae have received special attention due to their broad nutritional attributes, ecological benefits, and some species’ high digestibility, indicating that they have the potential to be used as a feed supplement in animal nutrition. Interestingly, some studies have revealed that microalgae have the potential to reduce methanogenesis in cattle, and the findings of this study demonstrated that indeed; the use of marine microalgae as a feed additive has positive effects on biogas, CH_4_, and H_2_S mitigation in sheep. Furthermore, microalgae can improve the characteristics of ruminal fermentation, and like other microalgae, *Dunaliella salina* is more effective in reducing the methanogenesis of high fibrous diets.

**Abstract:**

The aim of the present study was to evaluate the effects of marine microalgae (*Dunaliella salina*) as a food additive on biogas (BG), methane (CH_4_), carbon monoxide (CO), and hydrogen sulfide (H_2_S) production kinetics, as well as in in vitro rumen fermentation and the CH_4_ conversion efficiency of different genotypes of maize (*Zea mays* L.) and states of forage. The treatments were characterized by the forage of five maize genotypes (Amarillo, Montesa, Olotillo, Tampiqueño, and Tuxpeño), two states of forage (fresh and ensiled), and the addition of 3% (on DM basis) of microalgae (with and without). The parameters (*b* = asymptotic production, *c* = production rate, and *Lag* = delay phase before gas production) of the production of BG, CH_4_, CO, and H_2_S showed an effect (*p* < 0.05) of the genotype, the state of the forage, the addition of the microalgae, or some of its interactions, except for the time in the CO delay phase (*p* > 0.05). Moreover, the addition of microalgae decreased (*p* < 0.05) the production of BG, CH_4_, and H_2_S in most of the genotypes and stages of the forage, but the production of CO increased (*p* < 0.05). In the case of fermentation characteristics, the microalgae increased (*p* < 0.05) the pH, DMD, SCFA, and ME in most genotypes and forage states. With the addition of the microalgae, the fresh forage from Olotillo obtained the highest pH (*p* < 0.05), and the ensiled from Amarillo, the highest (*p* < 0.05) DMD, SCFA, and ME. However, the ensiled forage produced more (*p* < 0.05) CH_4_ per unit of SFCA, ME, and OM, and the microalgae increased it (*p* < 0.05) even more, and the fresh forage from Amarillo presented the highest (*p* < 0.05) quantity of CH_4_ per unit of product. In conclusion, the *D*. *salina* microalga showed a potential to reduce the production of BG, CH_4_, and H_2_S in maize forage, but its effect depended on the chemical composition of the genotype and the state of the forage. Despite the above, the energy value of the forage (fresh and ensiled) improved, the DMD increased, and in some cases, SCFA and ME also increased, all without compromising CH_4_ conversion efficiency.

## 1. Introduction

The human population is growing exponentially globally, and this trend is predicted to continue [1], leading to an increased demand for food, not only of plant origin but also of animal origin such as meat and milk [2]. Consequently, it is necessary to increase livestock production, which represents a challenge due to the instability that has occurred in recent years in environmental conditions, and the presence of extreme climatic phenomena such as heat waves, droughts, torrential rains, cyclones, hurricanes, and forest fires, among others [3,4]. Therefore, facing this challenge is not easy, and there are two ways to follow: extensification, which consists of expanding the area devoted to livestock, and intensification, which implies increasing production per surface unit. It is estimated that 80% of food will come from intensification, while the other 20% from extensification [5]. However, this entails an environmental cost that is inevitable, since livestock, especially ruminants, are an important source of greenhouse gases (GHG) [6,7]. So, increasing the number of livestock heads will increase the emission of GHG and their concentration in the atmosphere, which contributes to climate change.

GHG, also called biogas, can be produced throughout the ruminant digestive system, but most is produced in the rumen by ruminal microorganisms during feed degradation and fermentation [8,9]. Methane (CH_4_) and carbon dioxide (CO_2_) are the main GHG produced in ruminants, but other gases called trace gases are also generated, such as hydrogen (H_2_), carbon monoxide (CO), and hydrogen sulfide (H_2_S) [10]. Of these gases, CH_4_ is the most worrisome for livestock because its global warming potential is 28 times that of CO_2_, it has a half-life in the atmosphere of 8.6 years, and it represents an energy loss in animals [11]. However, the formation of CH_4_ is the main metabolic pathway to eliminate H_2_ and maintain the balance in the rumen for continuous feed degradation [12]. On the other hand, CO is an indirect GHG that increases the concentration of GHG, the utilization term of gases in the troposphere, and controls their movement into the stratosphere, which in turn affects the ozone layer [13,14]. Instead, the production of H_2_S is considered an alternate metabolic pathway for the elimination of H_2_ [15], but it is an odor gas, and in high concentrations, it is toxic for animals, which is why it causes adverse effects on the environment and livestock health [16]. Considering the need to produce more food and the environmental impact of livestock, it is essential to implement strategies to improve the efficiency of rumen fermentation and increase animal productivity, while mitigating GHG emissions [17] to direct livestock toward cleaner production.

In recent years, microalgae have received great attention from researchers from various countries, since it has been reported that they can be used as an additive/supplement in ruminant feed, either fresh, dry, or in extract form, because they present broad nutritional attributes and ecological benefits [18]. This is because they are sources of polyunsaturated fatty acids, proteins, polysaccharides, minerals, vitamins, photosynthetic pigments, antioxidants, and amino acids [19,20]. Furthermore, certain microalgae exhibit prominent feed digestibility, indicating their competence as a potent feed supplement [21]. Microalgal species such as *Schizochytrium* sp., *Chlorella* sp., *Arthrospira* sp., *Isochrysis* sp., and *Porphyridium* sp. have been used predominantly as feed supplements and have managed to improve livestock immunity, disease resistance, rumen microbial profile, intestinal function, and growth performance, among others [19]. Interestingly, certain studies have reported that the inclusion of microalgae in the ruminant diet can reduce the production of ruminal CH_4_ [22] and have attributed it to the content of bioactive compounds that they present, which act against the methanogenic population [23]. Other advantages that microalgae have, especially marine ones, is the ability to take advantage of solar radiation to convert CO_2_ into sugars and oxygen [18] and that they do not compete for land and freshwater surfaces, since they are produced in seawater that is more abundant than freshwater [24]. For this reason, unlike other investigations, this one was raised considering that maize forage (fresh, silage, or stubble) is one of the most common ingredients in ruminant diets [25] and that the positive effects of microalgae possibly depend on the state of the forage and the genotype. Therefore, the objective of the present study was to evaluate the effects of marine microalgae (*Dunaliella salina*) as a feed additive on the kinetics of biogas, CH_4_, CO, and H_2_S production, as well as on the characteristics of rumen fermentation in vitro and the CH_4_ conversion efficiency of different maize (*Zea mays* L.) genotypes and states of forage.

## 2. Materials and Methods

### 2.1. Experimental Treatments

The treatments were formed from the forage of five maize genotypes, two states of forage, and the addition of 3% (on DM basis) of marine microalgae, and a control treatment (without microalgae) was added per genotype and state of forage (Table 1). The evaluated maize genotypes were four natives of Mexico (Amarillo, Olotillo, Tampiqueño, and Tuxpeño) and one commercial hybrid (Montesa), all of them from a warm climate, and stand out for their high production and/or quality of forage. The species of marine microalgae evaluated was *D. salina*, and it is cultivated and produced by Allele Biotech de México, S. de R.L. de C.V. in the Ejido Zarahemla, Ensenada, Baja California, Mexico. The flour of this microalgae is a concentrate of marine microalgae, 100% natural and organic, grown in controlled ponds near the coastal zone.

### 2.2. Forage Production and Elaboration of Microsilages

The forage was produced in the municipality of Aldama, Tamaulipas, Mexico (22°59′09″ N and 98°10′25″ W, at 190 masl), under rainfed conditions between July and October 2021. The soil of the site has a loamy clay texture, with a high content of organic matter, and is moderately alkaline in nature, with low salinity. The climate, according to the Köppen classification, is of the Aw_0_ type, which corresponds to the driest of the warm subhumid ones [26]. The planting of each genotype was carried out in triplicate in 12 × 20 m plots (15 rows with a length of 20 m) and at a density of 62,500 plants ha^−1^. During the phenological cycle of the crop, pest and weed control was carried out manually, so pesticides or herbicides were not applied.

Before harvest, 10 representative plants from different points of each plot and genotype were selected, and they were cut 10 cm above ground level when the grain reached the milky-dough state. The plants from each plot and genotype were crushed separately, and a sample was obtained from the chopped forage from each plot, so there were a total of three samples per genotype, and they were called “fresh forage”. Meanwhile, with the rest of the forage, three 5 kg silages of each genotype were prepared, and for this, black polyethylene bags (30 cm diameter × 50 cm high, 500 caliber) were used, which were thermally sealed under vacuum. After 120 days, the silages were opened, and samples of fresh silage were obtained, which were dehydrated at 60 °C for 72 h and crushed in a hammer mill (Thomas Wiley^®^ Laboratory Mill model 4, Thomas Scientific^™^, Swedesboro, NJ, USA) with a 2 mm sieve.

### 2.3. Chemical Composition

Only the fresh and ensiled forage samples of the five maize genotypes were chemically analyzed, since the chemical composition of the marine microalgae was provided by the supplier. The analysis of the chemical composition of the fresh and ensiled forage included the content (%) of organic matter (OM) indirectly, by estimating the ashes (Ash) and subtracting the value obtained from 100 [27]; crude protein (CP), determining the amount of nitrogen [28] and multiplying the value obtained by 6.25; and neutral detergent fiber (NDF) and acid (ADF), with the methodology described by Van Soest et al. [29], using an ANKOM^200^ fiber analyzer (ANKOM Technology Corp., Macedon, NY, USA), acid detergent lignin (ADL) by solubilization with a sulfuric acid solution [30], ether extract (EE) following the method of Padmore [31], and nonfibrous carbohydrates (NFCs) and total carbohydrates (TCs) using the equations of Mertens [32] and Sniffen et al. [33]:

NFC = 100 − (CP + NDF + EE + Ash)(1)

TC = 100 − (CP + EE + Ash)(2)

### 2.4. In Vitro Incubation

The nutrient medium was prepared following the Goering and Van Soest [34] methodology, and the rumen fluid was obtained from four male sheep (40 ± 5 kg LW) slaughtered at the municipal slaughterhouse of Toluca, State of Mexico, Mexico. The rumen content was collected immediately after the animals were sacrificed, and it was transported to the laboratory in a hermetic thermos, where it was filtered with four layers of gauze and kept at 39 °C until use. The incubation was carried out in glass vials (160 mL), placing in each vial 500 mg of dehydrated sample of fresh forage or silage of each genotype, 50 mL of a solution containing nutrient medium and rumen fluid in a ratio of 4:1, and the percentage of corresponding microalgae, which for its application was diluted in 3 mL of distilled water. Each vial was hermetically sealed with butyl rubber stoppers and aluminum seals, and at the end, all were lightly shaken and incubated at 39 °C in an incubator (Binder^®^ BD series, BRINDER Inc., Tuttlingen, BaWü, Germany). Three incubation cycles were carried out, and in each cycle, all the treatments and the blank (without substrate and without microalgae) were incubated in triplicate.

#### 2.4.1. Measurement of Biogas, Methane, Carbon Monoxide, and Hydrogen Sulfide Production

Biogas (BG) production was measured in PSI (pound per square inch) at 2, 4, 6, 24, 26, 28, 30, and 48 h of incubation, following the technique of Theodorou et al. [35] and using a digital pressure manometer with an accuracy of ±2% (Manometer model 407910, Extech^®^ Instruments, Nashue, NH, USA). Similarly, methane (CH_4_), carbon monoxide (CO), and hydrogen sulfide (H_2_S) production were evaluated using the methodology proposed by Acosta et al. [36], which consists of extracting gas from the vials with a sterile plastic syringe, (Plastipak™, 5 mL 21 G × 32 mm; Becton Dickinson, Franklin Lakes, New Jersey, USA) and injecting it into a portable gas detector (Dräger X-am^®^, model 2500, Dräger, Lübeck, SH, Germany) by means of an external pump (Dräger X-am^®^, Dräger, Lübeck, SH, Germany). Once the measurement was completed in each reading, the biogas accumulated in the vials was released to avoid the partial dissolution of the gases [37].

#### 2.4.2. Ruminal Hydrogen Potential and Dry Matter Degradability

At the end of the incubation, the contents of the vials were filtered following the methodology of Alvarado-Ramírez et al. [38], which consists of retaining the residual forage in bags with a porosity of 25 µm (Filter bags F57, ANKOM Technology Corp., Macedon, NY, USA) and collecting the liquid in beakers. In the liquid, the hydrogen potential (pH) was measured with a potentiometer with a glass electrode (pH wireless electrode HALO^®^ model HI11102, Hanna^®^ Instruments, Woonsocket, RI, USA), while with the residual forage, the dry matter degradability was estimated by means of the difference between the weights of the forage at the beginning and at the end of incubation [39], so it was washed with plenty of water and dehydrated at 60 °C for 72 h.

#### 2.4.3. Calculations

The kinetics of the production of BG, CH_4_, CO, and H_2_S were estimated by adjusting the volume of the gases with the NLIN procedure of SAS [40], according to the model proposed by France et al. [41]:

y = *b* × [1 − e^−*c* (t − *Lag*)^] (3)

where

y = volume (mL) of BG, CH_4_, CO, and H_2_S at time t (h).

*b* = asymptotic BG, CH_4_, CO, and H_2_S production (mL g^−1^ DM).

*c* = rate of BG, CH_4_, CO, and H_2_S production (mL h^−1^).

*Lag* = initial delay time before BG, CH_4_, CO, and H_2_S production begins (h).

Metabolic energy (ME; MJ kg^−1^ DM) was estimated according to the equation proposed by Menke et al. [42]:

ME = 2.20 + (0.136 × PBG) + (0.057 × CP)(4)

where

PBG = net biogas production (mL 200 mg^−1^ DM) at 24 h of incubation.

CP = crude protein (g kg^−1^ DM).

Short-chain fatty acid (SCFA; mmol 200 mg^−1^ DM) concentrations were calculated according to Getachew et al. [43]:

SCFA = (0.0222 × PBG) − 0.00425(5)

where

PBG = net biogas production (mL 200 mg^−1^ DM) at 24 h of incubation.

Additionally, the ratios between CH_4_ and SCFA (CH_4_:SCFA; mmol mmol^−1^), ME (CH_4_:ME; g MJ^−1^), and OM (CH_4_:OM; mL g^−1^) were calculated.

### 2.5. Statistical Analysis

The statistical design was completely randomized with a trifactorial arrangement (5 × 2 × 2), where factor 1 was the maize genotype, factor 2 the state of the forage, and factor 3 the addition of the microalgae, and with three replications. The data from the three replicates of each treatment in each run were averaged, and the averages obtained were used as the experimental unit of each treatment. The data were analyzed using the GLM procedure of SAS [40] and the following statistical model:

Y_ijk_ = µ + G_i_ + S_j_ + M_k_ + (G × S)_ij_ + (G × M)_ik_ + (S × M)_jk_ + (G × S × M)_ijk_ + ε_ijk_ (6)

where Y_ijk_ is the response variable, μ is the general mean, G_i_ is the effect of the maize genotype, S_j_ is the effect of the state of the forage, M_k_ is the effect of the addition of microalgae, (G × S)_ij_ is the effect of the interaction between the maize genotype and the state of the forage, (G × M)_ik_ is the effect of the interaction between the maize genotype and the addition of microalgae, (S × M)_jk_ is the effect of the interaction between the state of the forage and microalgae addition, (G × S × M)_ijk_ is the effect of the interaction between maize genotype, state of the forage, and microalgae addition, and ε_ijk_ is the experimental error. A Tukey’s test was applied to compare means, and means with *p* ≤ 0.05 were considered statistically different from each other.

## 3. Results

The maize genotypes involved in the evaluation have a higher content of CP, NDF, and ADF in their fresh state, while in their ensiled state, they have a higher content of OM, EE, ADL, NFC, and TC (Table 2). As for the marine microalgae, it has a good content of proteins, as well as fulvic acids and minerals (Table 2).

### 3.1. Ruminal Biogas Production

**Figure 1:**
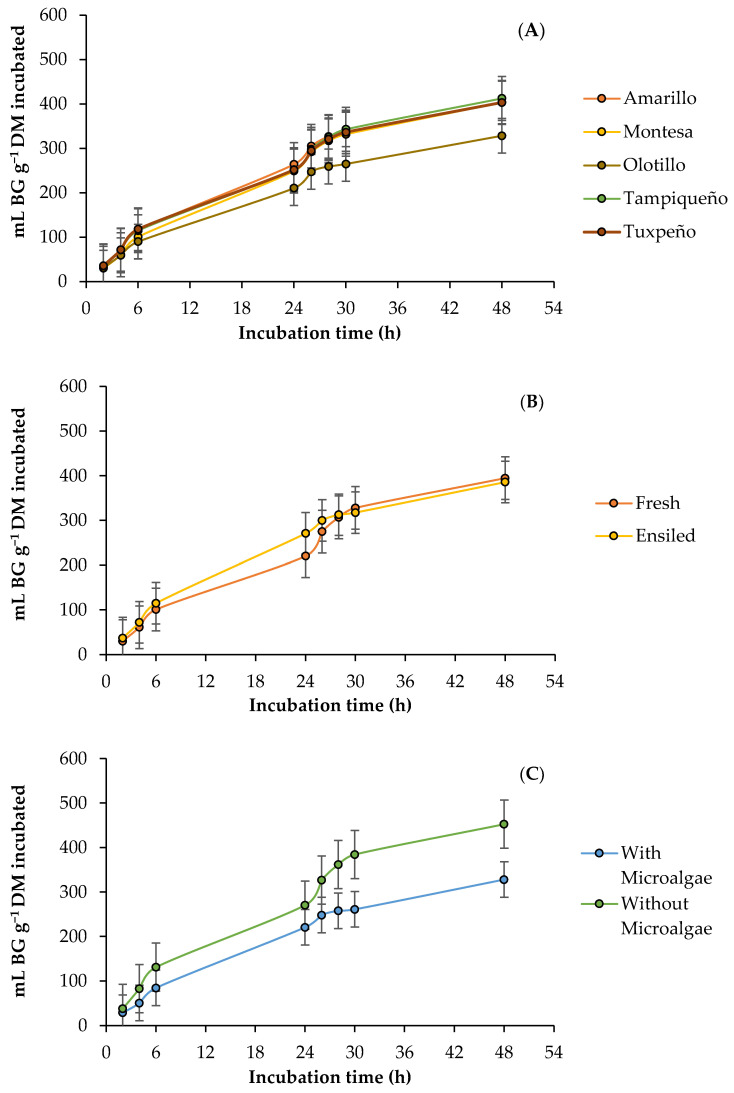
Kinetics of ruminal biogas (BG) production from maize forage in response to genotype: (**A**) Amarillo, Montesa, Olotillo, Tampiqueño, and Tuxpeño states of forage; (**B**) fresh or ensiled; (**C**) with or without *D. salina*.

The kinetics of the ruminal production of biogas (BG) by DM incubated from the fresh and ensiled forage of the evaluated maize genotypes, with and without the addition of the microalgae, are presented in Figure 1. The interactions genotype (G) × microalgae (M) and state (S) × M affected (*p* < 0.05) all parameters, and in the case of the BG production rate, the G × S interaction also affected (*p* < 0.05). Asymptotic production and lag phase time decreased with the addition of microalgae (4.4–39.0%) in all genotypes, and Amarillo and Tuxpeño presented the highest (352.0 mL g^−1^ DM and 1.93 h) and lowest (319.6 mL g^−1^ DM and 1.75 h) values, respectively. A similar effect was presented in the states of the forage, where the microalgae reduced asymptotic production and the time of the lag phase, and in both cases, fresh forage presented the greatest reduction (42.1 vs. 13.0%) and the lowest (306.5 mL g^−1^ DM and 1.68 h) values. In the production rate, the silage presented the highest rate in the Amarillo, Montesa, and Olotillo genotypes, while in Tampiqueño and Tuxpeño, it was in the fresh forage, and of all the genotypes, the Montesa silage presented the highest rate (0.0312 mL g^−1^ DM) and the fresh forage from Olotillo the lowest (0.0266 mL g^−1^ DM). However, the microalgae decreased (3.2 and 13.1%) the production rate of the Amarillo and Montesa genotypes, while that in Olotillo, Tampiqueño, and Tuxpeño increased (10.9, 13.9 and 16.4%), and of all the genotypes, Olotillo presented the highest (0.0300 mL g^−1^ DM) and the lowest (0.0248 mL g^−1^ DM) BG rate with and without the microalgae, respectively. In addition, without considering the genotype, the microalgae decreased (0.8%) the production rate of fresh forage and increased (10.4%) the rate of silage, and of both states, silage presented the highest production rate (0.0308 mL g^−1^ DM). In the production of BG, at 6 and 24 h, the interaction G × S × M influenced (*p* < 0.05), and the silage presented the highest production (125.55–321.54 mL g^−1^ DM) in all the genotypes, except in Amarillo and Montesa at 6 h, where the greatest accumulation was obtained by fresh forage (136.81 vs. 129.52 and 132.11 vs. 107.10 mL g^−1^ DM). In addition, the microalgae decreased (2.5–45.0%) the production of BG in both stages of the forage, and the silage maintained the highest production (216.47–290.68 mL g^−1^ DM). In contrast, at 48 h, they affected (*p* < 0.05) the interactions G × S, G × M, and S × M, and in all genotypes, fresh forage presented the highest production (411.61–446.49 mL g^−1^ DM), with the exception of Olotillo, and the addition of the microalgae decreased (4.8–39.7%) the production of BG in both states of the forage (Table 3).

### 3.2. Ruminal Methane Production

**Figure 2:**
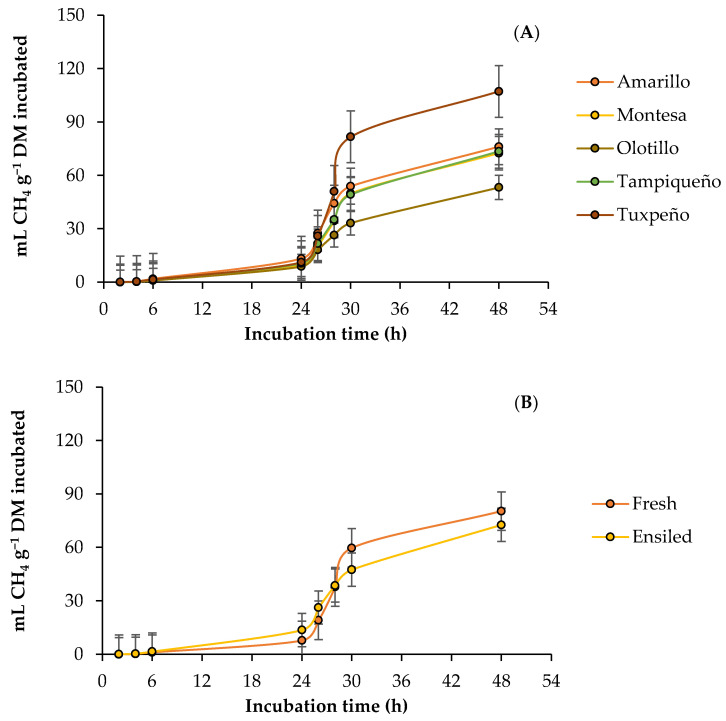

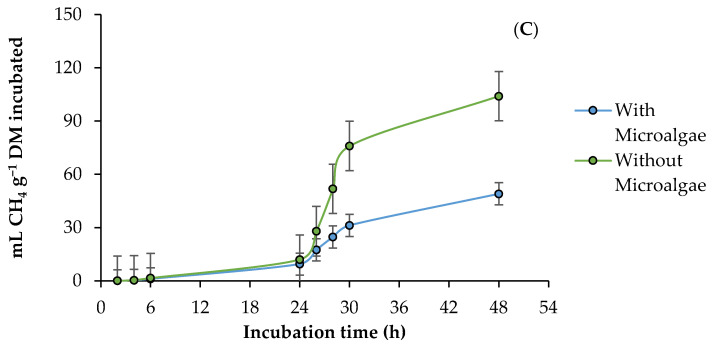
Kinetics of ruminal methane (CH_4_) production from maize forage in response to genotype: (**A**) Amarillo, Montesa, Olotillo, Tampiqueño, and Tuxpeño states of forage; (**B**) fresh or ensiled; (**C**) with or without *D. salina*.

The kinetics of the ruminal production of methane (CH_4_) by DM incubated from the fresh forage and silage of the evaluated maize genotypes, with and without the addition of the microalgae, are presented in Figure 2. The parameters of the CH_4_ production presented an effect (*p* < 0.05) of the G × S × M interaction, and without the microalgae, the fresh forage presented the highest asymptotic production (103.3–238.46 mL g^−1^ DM) and production rate (0.1504–0.2183 mL h^−1^), thus the longest time in the lag phase (17.89–41.3 h) in all genotypes, except in Olotillo. However, the addition of the microalgae decreased (2.4–81.8%) the value of the parameters, except the production rate of Olotillo in both states, and in all the genotypes, the silage presented the highest asymptotic production (48.53–76.65 mL g^−1^ DM), production rate (0.0781–0.0930 mL h^−1^), and time in the lag phase (5.92–13.28 h). In this case, the lowest asymptotic production (34.17 mL g^−1^ DM) and the shortest time in the lag phase (5.92 h) was obtained by the fresh forage from Olotillo with the microalgae, while the lowest production rate (0.0699 mL h^−1^) was presented by the fresh forage from Tuxpeño with the microalgae. In addition, the highest values (238.46 mL g^−1^ DM, 0.2183 mL h^−1^ and 41.3 h) in the parameters were obtained without the addition of the microalgae (Table 4). In CH_4_ production, the G × S × M interaction had an effect (*p* < 0.05) at 6 and 24 h, and although the microalgae decreased (8.58–73.23%) CH_4_ production in most of the genotypes and both states of the forage, the silage presented the highest production (0.88–17.86 mL g^−1^ DM) in both hours, with and without the microalgae. In addition, at 6 h, the highest production (3.1 mL g^−1^ DM) was obtained by the fresh forage from Amarillo without the microalgae and the lowest (0.7 mL g^−1^ DM) by the fresh forage from Montesa with the microalgae, while at 24 h, Montesa continued with the lowest production (5.41 mL g^−1^ DM), and the silage from Tampiqueño presented the highest (17.86 mL g^−1^ DM), in both cases without the microalgae. However, at 48 h, it only presented an effect (*p* < 0.05) of the S × M interaction, and without the addition of the microalgae, the highest CH_4_ production (107.9 mL g^−1^ DM) was obtained by the fresh forage, while the lowest (86.8 mL g^−1^ DM) was silage. However, with the addition of the microalgae, the production of CH_4_ decreased in both stages of the forage, and the silage obtained the highest production (58.54 vs 39.46 mL g^−1^ DM) compared to the fresh forage. In the proportion of CH_4_ (mL 100 mL^−1^ BG), there was also an effect (*p* < 0.05) of the interaction G × S × M, and although the microalgae increased the proportion at 6 h (13.61–74.19%), it decreased at 24 (8.89–35.90%) and 48 h (9.86–66.53%) in most genotypes and both states of the forage. In addition, the ensiled forage presented the highest proportion of CH_4_ in most of the genotypes during the entire incubation, with and without the addition of microalgae (Table 4).

### 3.3. Ruminal Carbon Monoxide Production

**Figure 3:**
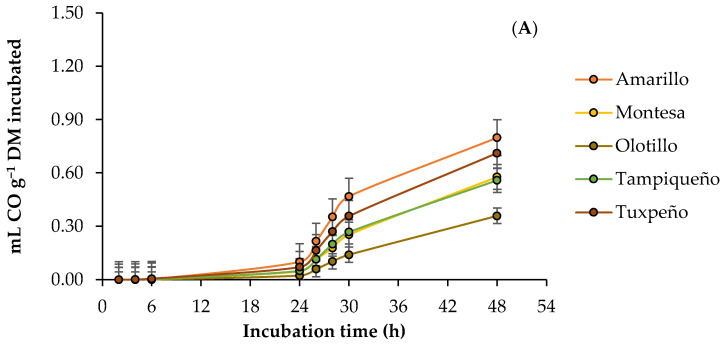

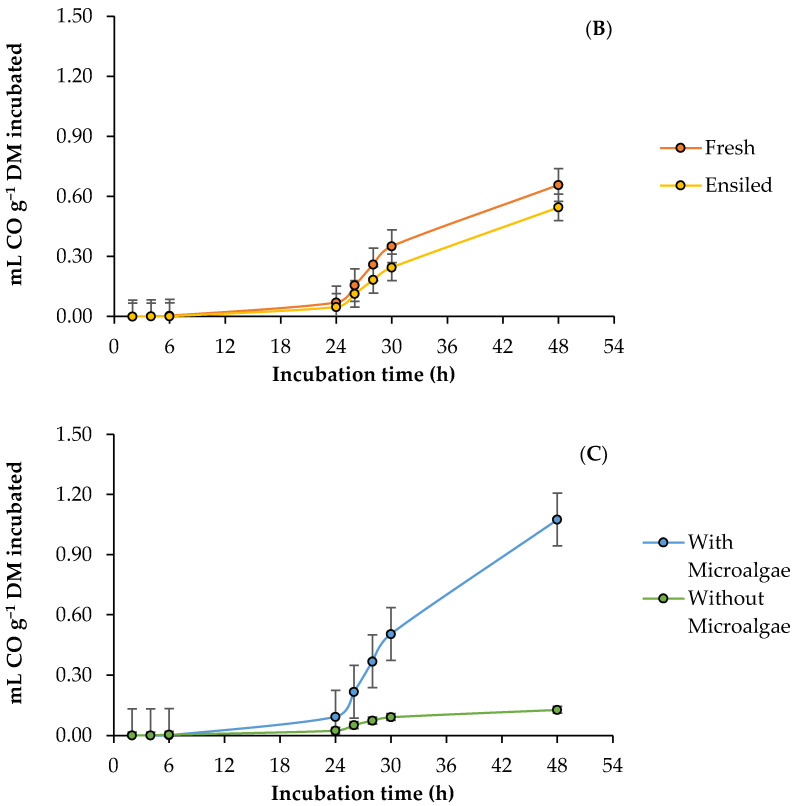
Kinetics of ruminal carbon monoxide (CO) production from maize forage in response to genotype: (**A**) Amarillo, Montesa, Olotillo, Tampiqueño, and Tuxpeño states of forage; (**B**) fresh or ensiled; (**C**), with or without *D. salina*.

The kinetics of the ruminal production of carbon monoxide (CO) by incubated DM of the fresh forage and silage of the corn genotypes evaluated, with and without the addition of microalgae, are presented in Figure 3. The asymptotic production presented an effect (*p* < 0.05) of the G × S × M interaction, and without the microalgae, the fresh forage obtained the highest production (0.0535–1.2500 mL g^−1^ DM) in all genotypes. However, the addition of the microalgae increased (6.20–18,281%) the asymptotic production in both states of the forage of all the genotypes, and the highest production in Olotillo and Tuxpeño was presented in the silage, while in the rest of the genotypes remained in the fresh forage. In addition, the fresh forage from Amarillo obtained the highest asymptotic production (4.7611 mL g^−1^ DM) with the microalgae, while without the microalgae, the silage from Olotillo had the lowest (0.0215 mL g^−1^ DM). The production rate was only influenced (*p* < 0.05) by the microalgae and increased from 0.0003 to 0.0033 mL h^−1^, while the time of the lag phase did not differ (*p* > 0.05) between genotypes, states, and with the microalgae. In the production of CO, at 6 h, the interaction G × S × M was significant (*p* < 0.05), and without the microalgae, all the genotypes presented the highest production in fresh forage (0.0029–0.0185 mL g^−1^ DM) except Olotillo. In addition, the highest production (0.0185 mL g^−1^ DM) was obtained by the fresh forage from Tuxpeño, and the lowest (0.0005 mL g^−1^ DM) by the fresh forage and silage from Olotillo. However, at 24 h, only the G × M interaction was affected (*p* < 0.05), and the addition of the microalgae increased (120.80–1365.09%) CO production in all genotypes. At this time, the highest production (0.1877 mL g^−1^ DM) was obtained by the Amarillo genotype with the microalgae, and the lowest (0.0064 mL CO g^−1^ DM) by Olotillo without the microalgae. In contrast, at 48 h, genotype and microalgae influenced (*p* < 0.05), and Amarillo obtained the highest production (0.7986 mL g^−1^ DM), while Olotillo the lowest (0.3590 mL g^−1^ DM). Furthermore, the addition of the microalgae increased the accumulated production from 0.1271 to 1.0756 mL g^−1^ DM (Table 5).

### 3.4. Ruminal Hydrogen Sulfide Production

**Figure 4:**
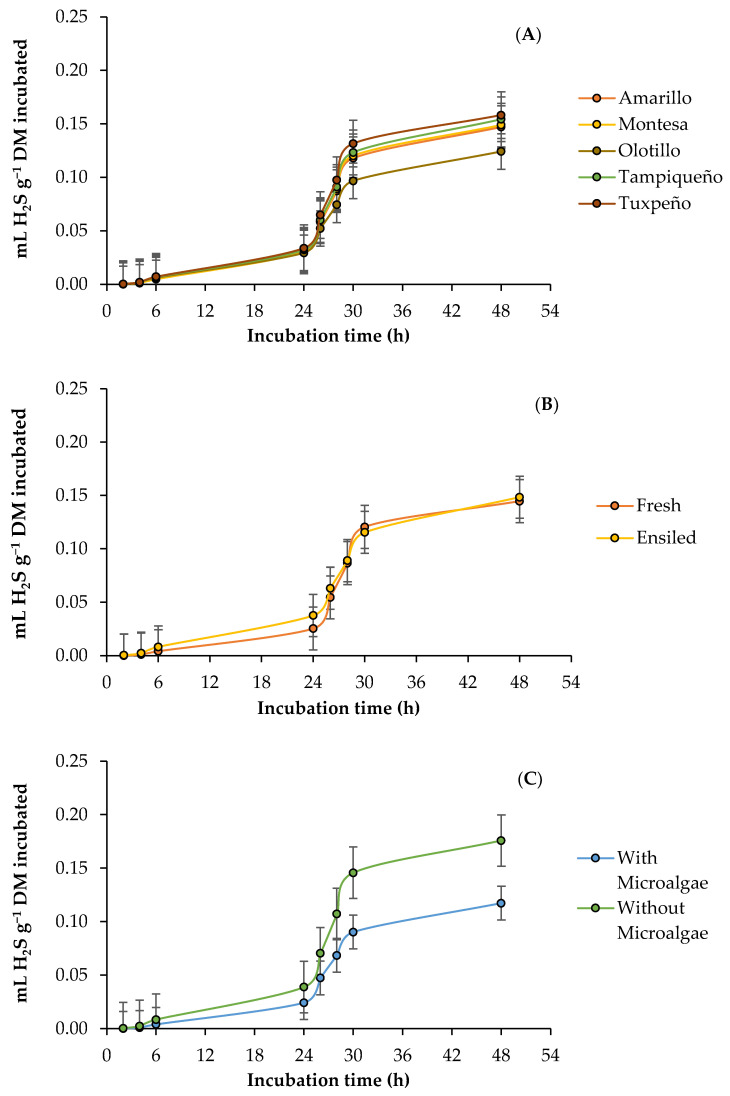
Kinetics of ruminal hydrogen sulfide (H_2_S) production from maize forage in response to genotype: (**A**) Amarillo, Montesa, Olotillo, Tampiqueño, and Tuxpeño states of forage; (**B**) fresh or ensiled; (**C**) with or without *D. salina*.

The kinetics of ruminal hydrogen sulfide (H_2_S) production by DM incubated from fresh and ensiled forage of the evaluated maize genotypes, with and without the addition of the microalgae, are presented in Figure 4. Regardless of the genotype and the state of the forage, the addition of the microalgae decreased (*p* < 0.05) the asymptotic production (0.1076 vs. 0.0511 mL g^−1^ DM), the production rate (0.00021 vs. 0.00016 mL h^−1^), and the time in the lag phase (0.0008 vs. 0.0004 h). In addition, the G × S interaction affected (*p* < 0.05) the production rate, so the highest rate in fresh forage occurred in Tampiqueño (0.00020 mL h^−1^) and Tuxpeño (0.00020 mL h^−1^), while in silage, it was in Amarillo (0.00020 mL h^−1^) and Olotillo (0.00019 mL h^−1^), and in Montesa, it was similar (0.00019 mL h^−1^) between forage states. However, the accumulated production of H_2_S presented an effect (*p* < 0.05) of the interaction G × S × M at 6 h of incubation, and it was observed that in all genotypes the silage forage obtained the highest accumulation (0.0079–0.0155 mL g^−1^ DM), and that in both stages of the forage, the addition of microalgae decreased (28–76%) the accumulation of H_2_S, except in Tampiqueño, in which it increased (8.3%). Although at 24 h the S × M interaction was significant (*p* < 0.05), the ensiled forage maintained the highest production compared to the fresh forage (0.0486 vs. 0.0292 mL g^−1^ DM), and in both cases, the addition of the microalgae decreased the production of H_2_S by 25.9 and 45.0%, respectively, for the fresh forage and silage. In the case of 48 h, the interactions G × S, G × M, and S × M showed an effect (*p* < 0.05) on the accumulation of H_2_S, and an effect similar to that of 6 and 24 h of incubation was observed, but with the difference that in the Amarillo and Olotillo genotypes, the highest accumulated production occurred with silage, and with the addition of microalgae, H_2_S production decreased by 41.0 and 24.9%, respectively, for the fresh forage and silage (Table 6).

### 3.5. Ruminal Fermentation Characteristics and CH_4_ Conversion Efficiency

The pH and the degradation of the dry matter (DMD) presented an effect (*p* < 0.05) of the G × S and S × M interactions, and in the case of the pH, it also presented an effect of the G × M interaction (*p* < 0.05). The fresh forage obtained the highest pH (7.07–7.32) in all genotypes, while the silage the lowest (6.91–7.10), and the fresh forage from Olotillo and Amarillo silage obtained the highest value (7.32) and lowest (6.91), respectively.

In addition, the microalgae reduced the pH of the Amarillo (7.01 vs. 6.96), Montesa (7.06 vs. 7.05), and Tuxpeño (7.10 vs. 7.06) genotypes, while in Olotillo (7.17 vs. 7.24) and Tampiqueño (7.14 vs. 7.17), increased it. Similarly, microalgae increased (7.16 vs. 7.19) the pH of fresh forage and decreased it (7.03 vs. 7.00) in silage. In the DMD, the silage (50.5–60.6%) presented the highest degradability in all the genotypes and the fresh forage the lowest (35.5–45.0%), but with the addition of microalgae, the degradation in both states of the forage improved, especially in silage, where it increased 41.5%. Short-chain fatty acid (SFCA) and metabolizable energy (ME) presented an effect (*p* < 0.05) of the G × S × M interaction. In this case, the microalgae increased SFCA (10.3–40.3%) and ME (3.4–11.2%) in both states of the forage of the Montesa, Olotillo, Tampiqueño, and Tuxpeño genotypes, except in the Olotillo silage, where SCFA (5.1–13.8%) and ME (1.6–5.4%) decreased, as well in both states of the Amarillo genotype with the addition of the microalgae. Similarly, the G × S × M interaction affected the CH_4_ conversion efficiency, and in the Amarillo, Montesa, and Tampiqueño genotypes, the microalgae increased CH_4_ per unit of SFCA (9.2–114.7%), ME (2.9–108.9%), and OM (10.2–105.7%) in both stages of the forage, with the exception of CH_4_ by SFCA in the fresh forage of Montesa, where it decreased (6.2%). Contrary to this, in both states of the Olotillo forage and the Tuxpeño silage, the microalgae reduced the amount of CH_4_ per unit of SFCA (42.6–60.5%), ME (27.6–59.5%), and OM (19.7–61.3%), although in the fresh forage of Tuxpeño, it increased by 143.3, 169.7, and 187.7%, respectively (Table 7).

## 4. Discussion

### 4.1. Ruminal Biogas Production

Biogas production is closely related to feed degradability and thus in turn with the fast-fermenting nutrients available for the activities and growth of the rumen microbiota [23]. However, BG is basically made up of CO_2_ and CH_4_, and the production of these gases depends mainly on the fermentation of carbohydrates to SCFA and proteins, although their contribution to BG is minor compared to the contribution of carbohydrates [44]. In addition, the formation of acetate and butyrate during rumen fermentation generates a greater amount of gas compared to the formation of propionate, so that acetate and butyrate contribute most of the BG [45]. This indicates that the high rate of BG production in the silage from Amarillo, Montesa, and Olotillo and the fresh forage from Tampiqueño and Tuxpeño was the result of a higher degradability or formation of acetate and butyrate, caused by the differences in the chemical composition of the genotypes and forage states. Similarly, variations in chemical composition are associated with the effect of microalgae, since microalgae can increase microbial activity during rumen fermentation when there is availability of easily fermentable nutrients [46]. Consequently, this decreases the time in the lag phase and increases the rate and asymptotic BG production of the genotypes and forage states with higher availability of fermentable nutrients, as occurred in the current study. In cases such as the Olotillo, Tampiqueño, Tuxpeño, and silage genotypes, where the microalgae increased the BG rate, it is possible that it was the consequence of a greater degradation or production of acetate and butyrate; as previously indicated, these are the SCFA that contribute the most gas to BG.

The addition of the microalgae reduced BG production in both stages of the forage of all genotypes in the current study, which is in line with what was reported by Elghandour et al. [23], who observed that the BG decreased with the addition of the microalgae *Schizochytrium* spp. and associated it with the antimicrobial and cytotoxic effects of the compounds of some microalgae [47], as well as the long-chain fatty acid profile [48]. Considering the above, it is likely that the microalgae have modified the structure of the microbial community during fermentation and that this caused variations in the final fermentation products, including the SCFA profile, as has been reported in other studies [49]. Furthermore, this motivates to assume that the BG reduction with the addition of microalgae was greater in fresh forage due to its antimicrobial action, since degradation was not affected and increased very little compared to silage (0.7 vs. 42.0%), which presented the highest production of BG. This high production of BG in the silage can be attributed to the higher availability of starch and percentage of NFC that it presents in comparison with fresh forage, since these carbohydrates constitute an immediate source of energy for the rumen microbiota [50]. The foregoing favors the fermentation and degradation of the feed [51] and was potentiated with the addition of microalgae, since the silage presented a greater increase in degradability compared to fresh forage. Regarding the slight variations in BG production during fermentation, they are attributed to the fact that NFC are made up of different types of carbohydrates (starch, sugars, fructans, and peptide substances) that vary in fermentability and degradation rate [52,53]. Therefore, it is feasible to assume that the microalgae were increasing the production of BG in response to the fermentability and digestibility of the carbohydrates that make up the NFC of each genotype and state of forage.

### 4.2. Ruminal Methane Production

Carbohydrates represent the main source of substrate for the formation of acetate and butyrate during ruminal fermentation, and as a by-product, CO_2_ and hydrogen (H_2_) are generated, which are used by methanogenic archaea for the production of CH_4_ [12]. In addition, despite the fact that maize forage (whole plant) has a high starch content, it is encapsulated by a protein matrix, which limits its digestion and utilization for the production of propionate [54]. Instead, ensiled forage goes through a fermentation process that generates a partial hydrolysis of the endosperm matrix proteins [51], allowing greater starch degradation by microbes and promoting a decrease in pH. In the rumen, this substrate may favor the production of propionate at the expense of acetate, reducing the population of protozoa and the H_2_ available for the production of CH_4_ [55], thus reducing CH_4_ accumulation.

With the addition of microalgae, in the fresh forage, the highest CH_4_ production was obtained in Amarillo and the lowest in Olotillo, while in the silage, they were Tampiqueño and Olotillo, and the accumulation of CH_4_ decreased in both states of forage. Some previous studies have indicated that the use of microalgae (*Chlorella* sp. and *Chlorella vulgaris*) as a strategy to mitigate CH_4_ production is more effective in diets based on forage or with a high content of fibrous carbohydrates [56,57], which is in agreement with the results found here. Although no studies have been reported on the mitigation of ruminal CH_4_ production using the microalga *D*. *salina* as an additive, studies with other microalgae (*Spirulina platensis*, *Chlorella vulgaris*, and *Schizochytrium* spp.) have reported the antimethanogenic effect observed in this study and attributed it to the content of docosahexaenoic acid (C22:6 n−3) and eicosapentaenoic acid (C20:5 n−3) that they present, since these polyunsaturated acids reduce the concentration of acetate and increase that of propionate and decrease the abundance of methanogenic archaea, the main CH_4_-producing microorganisms [23,45,58,59]. In addition to the above, Sheng et al. [60], reported that humic compounds, such as humic and fulvic acids, have the ability to reduce CH_4_ production in ruminants and attributed this to the reduction in the molar proportion of acetate and protozoa populations [61], which decreases the H_2_ available for the production of CH_4_ [62]. In addition, when evaluating three microalgae (*Spirulina platensis*, *Chlorella vulgaris*, and *Schizochytrium* spp.) as an additive in the feed of ruminants, Sucu [45] observed an increased proportion of propionate, suggesting a mitigation of methanogenesis given that propionate formation is an effective H_2_ sink.

### 4.3. Ruminal Carbon Monoxide Production

Despite the fact that carbon monoxide (CO) is considered an indirect greenhouse gas [14], there are few investigations that have been carried out on the production of CO in ruminant livestock [38,63]. However, it has been reported that under anaerobic conditions, CO is produced from the degradation of organic matter (OM) [64], and in the current study, without microalgae, the production of CO was mainly higher in the fresh forage of all the genotypes, while with the addition of microalgae, the accumulation increased in both stages of the forage, and the fresh forage continued to be the one with the highest production. The variation in CO between the states of forage without microalgae can be attributed to the chemical compounds that constitute the OM and its degradability, since it has been reported that the production of CO depends more on the chemical composition of the OM than on the quantity, as well as the ruminal microbiota [38,63]. In addition, the fresh forage also presented the highest CH_4_ production without the microalgae, indicating a greater H_2_ oxidation and CO_2_ reduction for the formation of CH_4_, and during these processes, CO is generated [65], which translates into a higher production of CO, which may be the reason why this forage presented the highest production. However, the production obtained with the addition of the microalgae was suggested to be caused by acetogenesis, since the inhibition of methanogenesis favors the increase of reducing acetogens [58], which increases the reduction of CO_2_ to CO. In addition, some carbon monoxide dehydrogenase enzymes involved in the Wood–Ljungdahl pathway during CO_2_ reduction are dependent on mineral availability [66], so the effect of the mineral profile of the microalgae cannot be disregarded (Table 2).

### 4.4. Ruminal Hydrogen Sulfide Production

Hydrogen sulfide (H_2_S) is produced mainly by sulfo-reducing bacteria during rumen digestion of feed, and although the amount of gas depends on the availability of sulfur (S) entering the rumen [67], there are several factors that affect the functions of the sulfo-reducing bacteria, and consequently the production of H_2_S. According to Zhao and Zhao [68], some of these factors are the activity of the sulfite reductase enzyme, fast-fermenting carbohydrates, nitrogen content, available minerals, and rumen pH. Considering this and that the S content in forage is not affected by silage [69], it is likely that the variations in the H_2_S production rate between genotypes and forage states were caused by differences in carbohydrate and nitrogen content. In addition, it is suggested that it was higher in the ensiled because silage favors the availability of nutrients from higher fermentation and lactate, a compound that is also used by sulfo-reducing bacteria as a substrate for the production of H_2_S [68]. On the other hand, the reduction in the parameters with the addition of the microalgae is attributed to an increase in the activity and growth of the rumen microbes, reflected in a shorter time in the delay phase, and to the antimicrobial effects that they exert on some groups of rumen microbes, including ciliated protozoa and methanogens [70], microbes that produce and consume H_2_, a gas used by sulfo-reducing bacteria for H_2_S production [71]. In this sense, it is known that the abundance of sulfo-reducing bacteria in the rumen is small (10^5^–10^6^ cell mL^−1^) compared to methanogens, a group of microbes that consume H_2_, and even so, they are able to compete for H_2_ when they have a high availability of S [72]. Therefore, the reduction in H_2_S in most genotypes and states of forage with the addition of microalgae can be attributed to changes in the microbial community structure caused by the antimicrobial effects of the microalgae, especially on the hydrogenogens and methanogens. Consequently, it is possible that H_2_ has been redirected toward other metabolic processes such as propriogenesis, especially in ensiled forage, which caused a reduction in the amount of H_2_ available [73], and by not having a source of S, the sulfo-reducing bacteria were dominated by methanogens, and consequently, the production of H_2_S decreased.

### 4.5. Ruminal Fermentation Characteristics and CH_4_ Conversion Efficiency

Ensilage allows the conservation of forage by lowering pH as result of a lower buffering capacity, caused by a greater availability of NFC, and the high content of lactic acid [74]. The addition of microalgae led to a reduction in pH in all genotypes and both stages of the forage that may be attributed to an increase in the production of SCFA, since the pH decreases with the increasing accumulation of SCFA [49]. Similarly, NFC also influenced DMD, since they are more fermentable than fibrous carbohydrates and provide immediate energy for the growth of the rumen microbiota [75]. However, carbohydrates are very diverse and differ in fermentability [53]. Furthermore, according to Kholif et al. [57], microalgae promote the fermentation of carbohydrates by rumen microbes, which is consistent with what was observed with the addition of microalgae and was attributed to the fulvic acids of the microalgae, since they can provide carbon to ruminal microorganisms [76] and thereby favor microbial growth and increase DMD. In turn, the increase in degradability was reflected in a greater production of SCFA and ME, which is attributed to a greater degradation of carbohydrates [57]. Although not evaluated in the present study, the increase in SFCAs and ME with the microalgae is possibly due to a greater activity of the fibrolytic bacteria [10] and an increase in the production of propionate, while in the cases where it decreased, it is attributed to the reduction in other SCFA such as acetate [48]. Meanwhile, the calculated variations in CH_4_ per unit of SCFA, ME, and OM may reflect the effects on DMD and SCFA, related to the composition and degradability of feed carbohydrates [77].

## 5. Conclusions

Under an in vitro rumen fermentation system, the addition of the marine microalga *D*. *salina* as a feed additive showed interactions with the maize genotype and the state of the forage for the production of BG, CH_4_, CO, and H_2_S, as well as for the characteristics of rumen fermentation and CH_4_ conversion efficiency. Although the microalgae increased CO, they showed the potential to reduce the production of BG, CH_4_, and H_2_S, as well as increase the DMD and, in some cases, improve the amount of SCFA and ME of the maize forage in all genotypes, without compromising CH_4_ conversion efficiency. Therefore, the use of microalgae as a feed additive in diets based on corn forage can contribute to reducing the environmental impact of livestock, without compromising animal productivity. However, it is necessary to carry out evaluations with different levels of addition of the microalgae and types of forage, as well as to carry out in vivo studies that allow maximizing the nutritional and environmental benefits that microalgae can provide.

## Figures and Tables

**Table 1:** Description of the experimental treatments.

Num.	Genotypes	States	Marine Microalgae ^1^
1	Amarillo	Fresh	Without
2			With
3		Ensiled	Without
4			With
5	Montesa	Fresh	Without
6			With
7		Ensiled	Without
8			With
9	Olotillo	Fresh	Without
10			With
11		Ensiled	Without
12			With
13	Tampiqueño	Fresh	Without
14			With
15		Ensiled	Without
16			With
17	Tuxpeño	Fresh	Without
18			With
19		Ensiled	Without
20			With

^1^ Without is the control treatment.

**Table 2:** Chemical composition (on DM basis) of the fresh and ensiled forage of different maize (*Zea mays* L.) genotypes and marine microalgae (*D. salina*).

Item ^1^	Genotypes of Maize ^2,3^										Marine Microalgae (*D. salina*) ^4^
	Amarillo		Montesa		Olotillo		Tampiqueño		Tuxpeño		
	FRE	ENS	FRE	ENS	FRE	ENS	FRE	ENS	FRE	ENS	
OM (%)	92.1	92.8	92.7	93.3	92.8	93.0	92.1	93.6	91.6	92.1	30.0–33.0
CP (%)	10.8	8.3	10.5	8.3	10.3	8.4	10.5	8.6	10.3	8.6	12.0–13.0
EE (%)	2.4	3.6	2.6	3.9	2.6	3.8	2.2	3.4	2.5	3.6	3.8
NDF (%)	59.7	47.6	52.6	50.4	66.2	59.8	61.7	59.5	58.9	52.9	-
ADF (%)	31.7	26.9	30.3	26.2	36.4	36.1	35.1	32.3	30.5	28.9	-
ADL (%)	3.8	4.1	3.7	3.9	4.4	4.9	4.3	4.9	3.7	4.2	-
NFC (%)	19.2	33.3	26.9	34.6	13.6	21.0	17.8	22.2	20.5	27.0	-
TC (%)	13.2	17.2	21.5	27.2	3.7	9.0	6.1	12.5	3.7	5.3	12.0–13.0
FV (%)											5.0–6.0
TN (%)											0.3–0.4
NH_3_ (%)											0.05–0.07
Nitrates (ppm)											50.0–60.0
Nitrites (ppm)											100.0–120.0
Phosphorus (%)											0.8–1.0
Potassium (%)											0.3–0.5
Calcium (%)											14.0
Magnesium (%)											8.0–9.0
Iron (ppm)											450.0–950.0
Boron (ppm)											200.0
Silica (ppm)											20.0
Copper (ppm)											10.0–15.0
Manganese (ppm)											15.0–20.0
Zinc (ppm)											10.0–15.0
Vanadium (ppm)											1.0–2.0

^1^ OM: organic matter; CP: crude protein; EE: ether extract; NDF: neutral detergent fiber; ADF: acid detergent fiber; ADL: acid detergent lignin; NFC: nonfibrous carbohydrate; TC: total carbohydrate; FV: fulvic acid; TN: total nitrogen; NH_3_: ammoniacal nitrogen. ^2^ FRE: fresh forage; ENS: ensiled forage. ^3^ The pH of the silages ranged between 3.6 and 3.8. ^4^ Analysis provided by the Allele Biotech de México, S. of R.L. of C.V., Ejido Zarahemla, Ensenada, B.C., Mexico.

**Table 3:** Parameters and cumulative ruminal biogas (BG) production by DM incubated from fresh and ensiled forage of different genotypes of maize (*Zea mays* L.), without and with addition of marine microalgae (*D. salina*), at 6, 24, and 48 h of incubation.

Genotypes	States	Marine Microalgae	BG Production					
			Parameters ^1^			mL BG g^−1^ DM Incubated		
			*b*	*c*	*Lag*	6 h	24 h	48 h
Amarillo	Fresh	Without	564.37	0.0283	3.09	136.81	265.16	526.81
		With	305.03	0.0268	1.67	83.12	203.23	296.41
	Ensiled	Without	398.20	0.0302	2.18	129.52	298.08	396.64
		With	398.90	0.0309	2.18	113.81	290.68	396.05
Montesa	Fresh	Without	582.63	0.0285	3.19	132.11	255.39	540.31
		With	303.23	0.0262	1.66	75.83	193.08	292.32
	Ensiled	Without	394.83	0.0301	2.16	107.10	273.02	388.15
		With	396.57	0.0322	2.17	89.09	275.05	392.98
Olotillo	Fresh	Without	304.13	0.0213	1.66	95.04	183.04	282.48
		With	288.90	0.0263	1.58	67.96	159.76	273.65
	Ensiled	Without	396.07	0.0283	2.17	125.55	283.41	390.61
		With	380.80	0.0336	2.08	71.73	216.47	367.02
Tampiqueño	Fresh	Without	616.13	0.0316	3.37	115.00	248.92	574.06
		With	322.67	0.0301	1.77	83.10	225.63	318.92
	Ensiled	Without	449.90	0.0250	2.46	168.40	312.37	436.93
		With	327.40	0.0286	1.79	92.73	222.43	320.68
Tuxpeño	Fresh	Without	579.07	0.0285	3.17	141.82	265.05	538.93
		With	312.60	0.0277	1.71	78.77	203.17	303.12
	Ensiled	Without	463.70	0.0258	2.54	161.37	321.54	452.19
		With	326.63	0.0286	1.79	90.33	217.55	319.59
Pooled SEM ^2^			42.523	0.00093	0.23264	6.139	16.267	39.330
*p*-value								
Genotype			0.0385	0.0166	0.0385	<0.0001	0.0005	0.0245
State			0.2036	<0.0001	0.2037	<0.0001	<0.0001	0.6269
Microalgae			<0.0001	0.0024	<0.0001	<0.0001	<0.0001	<0.0001
Genotype × State			0.0579	<0.0001	0.0579	0.0030	0.2391	0.0462
Genotype × Microalgae			0.0190	0.0010	0.0190	0.0026	0.1727	0.0262
State × Microalgae			<0.0001	0.0005	<0.0001	0.9350	0.6471	0.0002
Genotype × State × Microalgae			0.1554	0.3778	0.1554	<0.0001	0.0144	0.1361

^1^ *b*: asymptotic BG production (mL BG g^−1^ DM); *c*: rate of BG production (mL BG h^−1^); *Lag*: initial delay before BG production begins (h). ^2^ SEM: standard error of the mean.

**Table 4:** Parameters and cumulative ruminal methane (CH_4_) production by DM incubated and by 100 mL of BG from fresh and ensiled forage of different genotypes of maize (*Zea mays* L.), without and with the addition of marine microalgae (*D. salina*), at 6, 24, and 48 h of incubation.

Genotypes	States	Marine Microalgae	CH_4_ Production								
			Parameters ^1^			mL CH_4_ g^−1^ DM Incubated			mL CH_4_ 100 mL^−1^ BG		
			*b*	*c*	*Lag*	6 h	24 h	48 h	6 h	24 h	48 h
Amarillo	Fresh	Without	103.30	0.1504	17.89	3.10	13.46	105.10	2.27	5.07	19.92
		With	37.33	0.0791	6.47	0.83	6.62	37.29	1.00	3.25	12.57
	Ensiled	Without	85.01	0.0886	14.72	1.40	16.20	85.28	1.08	5.43	21.50
		With	76.65	0.0865	13.28	2.10	16.60	76.75	1.83	5.70	19.38
Montesa	Fresh	Without	109.29	0.1740	18.93	0.77	5.41	108.80	0.58	2.11	20.23
		With	37.63	0.0738	6.52	0.67	7.48	37.56	0.88	3.87	12.78
	Ensiled	Without	80.01	0.0884	13.86	1.14	13.87	80.18	1.07	5.08	20.67
		With	63.42	0.0787	10.98	1.23	12.68	63.42	1.38	4.60	16.10
Olotillo	Fresh	Without	50.16	0.0753	8.69	0.84	7.81	50.10	0.88	4.28	17.72
		With	34.17	0.0771	5.92	0.86	5.86	34.10	1.27	3.67	12.47
	Ensiled	Without	77.96	0.0867	13.50	1.28	12.82	78.20	1.02	4.50	19.95
		With	50.47	0.0930	8.74	0.88	8.89	50.59	1.22	4.10	13.77
Tampiqueño	Fresh	Without	106.85	0.1539	18.51	0.73	5.78	106.74	0.62	2.27	19.44
		With	45.09	0.0776	7.81	0.90	8.47	45.06	1.08	3.75	14.15
	Ensiled	Without	93.37	0.08540	16.17	2.47	17.86	93.63	1.47	5.72	21.43
		With	48.53	0.0781	8.41	1.55	10.21	48.52	1.67	4.60	15.13
Tuxpeño	Fresh	Without	138.46	0.2183	11.30	1.78	9.13	138.88	1.30	3.72	25.76
		With	43.45	0.0699	7.53	0.95	8.24	43.28	1.20	4.05	14.28
	Ensiled	Without	96.20	0.0832	16.66	2.10	17.70	96.54	1.30	5.50	21.32
		With	53.37	0.0813	9.24	1.37	9.72	53.43	1.52	4.47	16.70
Pooled SEM ^2^			18.618	0.00996	3.225	0.141	0.926	19.093	0.102	0.308	1.948
*p*-value											
Genotype			0.0044	0.0028	0.0044	<0.0001	<0.0001	0.1224	<0.0001	0.0008	<0.0001
State			0.3380	<0.0001	0.3380	<0.0001	<0.0001	0.9040	<0.0001	<0.0001	0.9761
Microalgae			<0.0001	<0.0001	<0.0001	<0.0001	<0.0001	<0.0001	0.0026	0.2425	<.0001
Genotype × State			0.0180	0.0001	0.0180	<0.0001	0.1372	0.3775	<0.0001	0.0016	0.0001
Genotype × Microalgae			0.0074	<0.0001	0.0074	0.0005	0.0100	0.2282	0.0006	0.0173	0.0005
State × Microalgae			0.0024	<0.0001	0.0024	0.0085	0.0006	0.0234	0.0002	0.0072	0.0014
Genotype × State × Microalgae			0.0406	0.0004	0.0406	<0.0001	<0.0001	0.4401	<0.0001	<0.0001	0.0002

^1^ *b*: asymptotic BG production (mL BG g^−1^ DM); *c*: rate of BG production (mL BG h^−1^); *Lag*: initial delay before BG production begins (h). ^2^ SEM: standard error of the mean.

**Table 5:** Parameters and cumulative ruminal carbon monoxide (CO) production by DM incubated from fresh and ensiled forage of different genotypes of maize (*Zea mays* L.), without and with the addition of marine microalgae (*D. salina*), at 6, 24, and 48 h of incubation.

Genotypes	States	Microalgae	CO Production					
			Parameters ^1^			mL CO g^−1^ DM Incubated		
			*b*	*c*	*Lag*	6 h	24 h	48 h
Amarillo	Fresh	Without	0.6273	0.0001	0.0008	0.0029	0.0209	0.1411
		With	4.7611	0.0070	0.0064	0.0007	0.1999	1.6132
	Ensiled	Without	0.0763	0.0001	0.0001	0.0007	0.0066	0.0414
		With	1.9180	0.0064	0.0518	0.0024	0.1755	1.3986
Montesa	Fresh	Without	0.7742	0.0011	0.0076	0.0054	0.0354	0.2078
		With	5.7051	0.0056	0.0010	0.0007	0.0529	0.7300
	Ensiled	Without	0.0224	0.0004	0.0000	0.0006	0.0069	0.0454
		With	1.7999	0.0007	0.9307	0.0013	0.0954	1.3290
Olotillo	Fresh	Without	0.0535	0.0000	0.0001	0.0005	0.0074	0.0356
		With	0.2461	0.0006	0.0048	0.0007	0.0406	0.7284
	Ensiled	Without	0.0215	0.0001	0.0000	0.0005	0.0055	0.0404
		With	1.0734	0.0089	0.0014	0.0013	0.0388	0.6317
Tampiqueño	Fresh	Without	0.1236	0.0009	0.0002	0.0034	0.0210	0.1465
		With	1.8035	0.0011	0.0024	0.0009	0.1275	1.3743
	Ensiled	Without	0.0277	0.0002	0.0000	0.0010	0.0059	0.0408
		With	0.3877	0.0002	0.2600	0.0024	0.0448	0.6751
Tuxpeño	Fresh	Without	1.2500	0.0001	1.3104	0.0185	0.1245	0.5351
		With	1.3275	0.0016	0.0053	0.0010	0.0656	1.0586
	Ensiled	Without	0.0259	0.0001	0.0000	0.0008	0.0048	0.0371
		With	4.7608	0.0013	0.0064	0.0026	0.0906	1.2169
Pooled SEM ^2^			1.04294	0.00246	0.35748	0.00112	0.02611	0.18557
*p*-value								
Genotype			0.0669	0.4891	0.6193	<0.0001	0.0024	0.0210
State			0.1674	0.9815	0.9561	0.0001	0.0660	0.1870
Microalgae			0.0243	0.0088	0.9757	0.0002	<0.0001	<0.0001
Genotype × State			0.1361	0.3791	0.2914	<0.0001	0.4407	0.2278
Genotype × Microalgae			0.0332	0.3630	0.2949	<0.0001	0.0010	0.0711
State × Microalgae			0.0722	0.7781	0.1187	<0.0001	0.2443	0.4680
Genotype × State × Microalgae			0.0169	0.4836	0.6176	<0.0001	0.0608	0.0676

^1^ *b*: asymptotic BG production (mL BG g^−1^ DM); *c*: rate of BG production (mL BG h^−1^); *Lag*: initial delay before BG production begins (h). ^2^ SEM: standard error of the mean.

**Table 6:** Parameters and cumulative ruminal hydrogen sulfide (H_2_S) production by DM incubated from fresh and ensiled forage of different genotypes of maize (*Zea mays* L.), without and with the addition of marine microalgae (*D. salina*), at 6, 24, and 48 h of incubation.

Genotypes	States	Marine Microalgae	H_2_S Production					
			Parameters ^1^			mL H_2_S g^−1^ DM Incubated		
			*b*	*c*	*Lag*	6 h	24 h	48 h
Amarillo	Fresh	Without	0.0907	0.00019	0.0007	0.0040	0.0237	0.1678
		With	0.0395	0.00014	0.0003	0.0027	0.0195	0.1082
	Ensiled	Without	0.1230	0.00023	0.0009	0.0112	0.0530	0.1776
		With	0.0590	0.00017	0.0004	0.0071	0.0312	0.1348
Montesa	Fresh	Without	0.1138	0.00011	0.0008	0.0049	0.0331	0.2128
		With	0.0347	0.00014	0.0003	0.0023	0.0170	0.0942
	Ensiled	Without	0.0951	0.00021	0.0007	0.0079	0.0443	0.1522
		With	0.0530	0.00017	0.0004	0.0045	0.0268	0.1364
Olotillo	Fresh	Without	0.0618	0.00026	0.0005	0.0066	0.0261	0.0910
		With	0.0614	0.00013	0.0005	0.0047	0.0249	0.1072
	Ensiled	Without	0.1080	0.00023	0.0008	0.0091	0.0453	0.1669
		With	0.0423	0.00015	0.0003	0.0040	0.0217	0.1317
Tampiqueño	Fresh	Without	0.0741	0.00025	0.0006	0.0036	0.0253	0.2122
		With	0.0530	0.00012	0.0004	0.0039	0.0256	0.1232
	Ensiled	Without	0.1311	0.00019	0.0010	0.0145	0.0513	0.1638
		With	0.0620	0.00014	0.0005	0.0046	0.0269	0.1179
Tuxpeño	Fresh	Without	0.1480	0.00020	0.0011	0.0070	0.0379	0.2263
		With	0.0470	0.00018	0.0004	0.0024	0.0212	0.1037
	Ensiled	Without	0.1303	0.00011	0.0010	0.0155	0.0492	0.1871
		With	0.0591	0.00014	0.0004	0.0036	0.0272	0.1156
Pooled SEM ^2^			0.01702	0.000020	0.00013	0.00100	0.00412	0.01778
*p*-value								
Genotype			0.2252	0.1262	0.2242	0.0412	0.6025	0.0847
State			0.0758	0.1452	0.0776	<0.0001	<0.0001	0.6414
Microalgae			<0.0001	<0.0001	<0.0001	<0.0001	<0.0001	<0.0001
Genotype × State			0.5024	0.0496	0.5099	0.0036	0.2079	0.0304
Genotype × Microalgae			0.2656	0.0520	0.2610	0.0021	0.6565	0.0215
State × Microalgae			0.4399	0.2361	0.4510	<0.0001	0.0004	0.0477
Genotype × State × Microalgae			0.1506	0.0729	0.1542	0.0125	0.1943	0.0577

^1^ *b*: asymptotic BG production (mL BG g^−1^ DM); *c*: rate of BG production (mL BG h^−1^); *Lag*: initial delay before BG production begins (h). ^2^ SEM: standard error of the mean.

**Table 7:** Ruminal fermentation characteristics and CH_4_ conversion efficiency of fresh and ensiled forage of different maize (*Zea mays* L.) genotypes, without and with the addition of marine microalgae (*D. salina*).

Genotypes	States	Marine Microalgae	Ruminal Fermentation Characteristics ^1^				CH_4_ Conversion Efficiency ^2^		
			pH	DMD	SCFA	ME	CH_4_:SCFA	CH_4_:ME	CH_4_:OM
Amarillo	Fresh	Without	7.09	39.42	3.73	5.59	33.63	3.61	4.74
		With	7.04	39.88	3.54	5.50	72.20	7.54	9.75
	Ensiled	Without	6.93	51.62	5.34	6.08	69.61	9.60	13.41
		With	6.88	69.59	5.05	5.93	111.39	15.10	20.68
Montesa	Fresh	Without	7.15	35.39	3.56	5.47	79.59	8.23	10.40
		With	7.14	35.60	4.11	5.75	86.90	10.01	13.44
	Ensiled	Without	6.97	47.12	4.67	5.74	40.15	5.25	6.98
		With	6.95	66.42	5.51	6.17	37.66	5.40	7.69
Olotillo	Fresh	Without	7.25	45.14	2.68	4.98	60.01	5.18	6.00
		With	7.38	44.78	3.76	5.54	34.45	3.75	4.82
	Ensiled	Without	7.09	44.30	4.21	5.51	148.36	18.26	23.31
		With	7.10	60.58	3.63	5.21	64.80	7.39	9.03
Tampiqueño	Fresh	Without	7.20	40.55	3.70	5.54	72.18	7.82	10.17
		With	7.28	41.53	4.08	5.73	121.30	14.47	19.95
	Ensiled	Without	7.08	39.23	3.62	5.23	35.28	3.92	4.71
		With	7.06	61.68	4.43	5.65	45.07	5.68	7.39
Tuxpeño	Fresh	Without	7.10	40.28	4.23	5.78	37.71	4.39	5.91
		With	7.09	40.41	5.08	6.21	91.74	11.84	17.00
	Ensiled	Without	7.10	47.88	3.69	5.41	101.72	11.10	14.07
		With	7.02	67.37	4.34	5.74	40.16	4.84	6.51
Pooled SEM ^3^			0.130	2.806	0.361	0.185	4.041	0.525	1.029
*p*-value									
Genotype			0.0015	0.1390	0.0005	0.0005	0.0009	<0.0001	<0.0001
State			<0.0001	<0.0001	<0.0001	<0.0001	<0.0001	<0.0001	<0.0001
Microalgae			<0.0001	<0.0001	<0.0001	<0.0001	0.2525	<0.0001	<0.0001
Genotype × State			0.0016	0.0016	0.2391	0.2391	0.0016	0.0266	0.1372
Genotype × Microalgae			0.0345	0.9176	0.1728	0.1728	0.0175	0.0094	0.0100
State × Microalgae			<0.0001	<0.0001	0.6472	0.6472	0.0074	0.0008	0.0006
Genotype × State × Microalgae			0.7751	0.9784	0.0144	0.0144	<0.0001	<0.0001	<0.0001

^1^ pH: ruminal pH; DMD: dry matter degradability (%); SCFA: short-chain fatty acid (mmol g^−1^ DM) at 24 h of incubation; ME: metabolizable energy (MJ kg^−1^ DM) at 24 h of incubation. ^2^ CH_4_:SCFA, methane:short-chain fatty acid ratio (mmol mmol^−1^) at 24 h of incubation; CH_4_:ME, methane:metabolizable energy ratio (g MJ^−1^) at 24 h of incubation; CH_4_:OM, methane:organic matter ratio (mL g^−1^). ^3^ SEM: standard error of the mean.

## Data Availability

The data presented in this study are available on request from the corresponding author.
